# Add-On Effect of Selenium and Vitamin D Combined Supplementation in Early Control of Graves’ Disease Hyperthyroidism During Methimazole Treatment

**DOI:** 10.3389/fendo.2022.886451

**Published:** 2022-06-15

**Authors:** Daniela Gallo, Lorenzo Mortara, Giovanni Veronesi, Simona AM Cattaneo, Angelo Genoni, Matteo Gallazzi, Carlo Peruzzo, Paolo Lasalvia, Paola Moretto, Antonino Bruno, Alberto Passi, Andrea Pini, Andrea Nauti, Maria Antonietta Lavizzari, Michele Marinò, Giulia Lanzolla, Maria Laura Tanda, Luigi Bartalena, Eliana Piantanida

**Affiliations:** ^1^ Endocrine Unit, Department of Medicine and Surgery, University of Insubria, Azienda Socio Sanitaria Territoriale (ASST) dei Sette Laghi, Varese, Italy; ^2^ Immunology and General Pathology Laboratory, Department of Biotechnology and Life Sciences, University of Insubria, Varese, Italy; ^3^ Research Center in Epidemiology and Preventive Medicine (EPIMED), University of Insubria, Varese, Italy; ^4^ Immuno-hematology and Transfusion Medicine, ASST dei Sette Laghi, Varese, Italy; ^5^ Laboratory of Microbiology, Department of Biotechnology and Life Science, University of Insubria, Varese, Italy; ^6^ Occupational, Preventive and Toxicology Unit, ASST Sette Laghi, Varese, Italy; ^7^ Department of Medicine and Surgery, University of Insubria, Varese, Italy; ^8^ Istituto di Ricovero e Cura a Carattere Scientifico (IRCCS) MultiMedica, Milan, Italy; ^9^ Laboratory of Clinical Chemical Analysis, ASST dei Sette Laghi, Varese, Italy; ^10^ Department of Clinical and Experimental Medicine, Endocrinology Unit II, University of Pisa and University Hospital of Pisa, Pisa, Italy

**Keywords:** Graves’ disease, hyperthyroidism treatment, vitamin D, selenium, quality of life

## Abstract

Prompt and stable control of hyperthyroidism is fundamental to avoid the detrimental effects of thyroid hormone excess, and antithyroid drugs, mainly methimazole (MMI), represent the first-line treatment for Graves’ disease (GD) hyperthyroidism. Decreased serum concentrations of selenium (Se) and calcifediol (25(OH)D, VitD) have been reported in newly diagnosed GD patients in observational studies. Low Se levels might exacerbate oxidative stress by compromising the antioxidant machinery’s response to reactive oxygen species, and low VitD levels might hamper the anti-inflammatory immune response. We performed a randomized controlled clinical trial (EudraCT 2017-00505011) to investigate whether Se and cholecalciferol (VitD) addition to MMI is associated with a prompter control of hyperthyroidism. Forty-two consecutive patients with newly-onset GD and marginal/insufficient Se and VitD levels were randomly assigned to treatment with either MMI monotherapy or MMI combined with Se and VitD. Se treatment was withdrawn after 180 days, while the other treatments were continued. Combination therapy resulted in a significantly greater reduction in serum FT4 concentration at 45 days (-37.9 pg/ml, CI 95%, -43.7 to -32.2 pg/ml) and 180 days (-36.5 pg/ml, CI 95%, -42 to -30.9 pg/ml) compared to MMI monotherapy (respectively: -25.7 pg/ml, CI 95%, -31.6 to -19.7 pg/ml and -22.9 pg/ml, CI 95%, -28 to -17.3 pg/ml, p 0.002). Data at 270 days confirmed this trend (-37.8 pg/ml, CI 95%, -43.6 to -32.1 pg/ml vs -24.4 pg/ml, CI 95%, -30.3 to -18.4 pg/ml). The quality of life (QoL) score was investigated by the validated “Thyroid-related Patient-Reported Outcome” questionnaire (ThyPRO). ThyPRO composite score showed a greater improvement in the intervention group at 45 days (-14.6, CI 95%, -18.8 to -10.4), 180 (-9, CI 95%, -13.9 to -4.2) and 270 days (-14.3, CI 95%, -19.5 to -9.1) compared to MMI group (respectively, -5.2, CI 95%, -9.5 to -1; -5.4, CI 95%, -10.6 to -0.2 and -3.5, CI 95%, -9 to -2.1, p 0-6 months and 6-9 months <0.05). Our results suggest that reaching optimal Se and VitD levels increases the early efficacy of MMI treatment when Se and VitD levels are suboptimal.

## Introduction

Graves’ disease (GD) is the most frequent cause of hyperthyroidism in iodine-replete geographical areas (1). Current management of Graves’ hyperthyroidism is largely imperfect, in the absence of therapies targeting the pathogenetic mechanisms of the disease (2, 3). Thionamide antithyroid drug (ATD) therapy is the first-line treatment worldwide under most circumstances (3, 4), but its major limitation is the high rate of relapses after drug discontinuation (3). With a few exceptions (e.g., first trimester of pregnancy), methimazole (MMI) is the preferred thionamide. Rapid and stable restoration of euthyroidism is fundamental because uncontrolled hyperthyroidism represents a cardiovascular risk (5).

Decreased serum concentrations of selenium (Se) and vitamin D (VitD) have been reported in newly diagnosed GD patients in observational studies (6–13). This could be relevant because Se, once integrated into selenoproteins (e.g., glutathione peroxidase), promotes thyrocyte defense against reactive oxygen species (ROS), the production of which is increased in hyperthyroidism (11–13). Severe hyperthyroidism has a deleterious impact on the activity of selenoproteins and Se availability, owing to increased Se demand along with decreased gastrointestinal absorption (12–14). It has been postulated that low Se levels might exacerbate oxidative stress in hyperthyroidism by compromising the antioxidant machinery’s response to ROS.

VitD, on the other hand, affects the maturation and differentiation of cells of the innate and adaptive immune systems, including dendritic cells, macrophages, natural killer cells and T cells subsets, thereby promoting anti-inflammatory and tolerogenic phenotypes (15, 16). Low VitD levels might increase the risk of developing autoimmune thyroid disorders or having a worse treatment response (15, 16). Although a rationale for adding Se or VitD to ATD exists based on the above findings, the combination of MMI with either Se or VitD in the management of Graves’ hyperthyroidism has provided conflicting results, at least partially explained by different baseline Se and VitD levels (17–28). This randomized controlled trial (RCT) sought to determine if concurrent supplementation with Se and VitD in Graves’ patients with suboptimal or frankly low Se and VitD levels may improve early control of hyperthyroidism during MMI treatment.

## Materials and Methods

### Subjects

From March 2019 to February 2020, at the Endocrine Unit in Varese, consecutive Caucasian patients with newly diagnosed GD, aged 18-70 years, in good health (except for hyperthyroidism) and able to provide written informed consent were screened for circulating calcidiol [25(OH)D, VitD] and Se levels. GD diagnosis was based on established criteria (2), including suppressed serum thyrotropin (TSH) and increased serum free thyroxine (FT4) and/or free triiodothyronine (FT3) levels, positive TSH receptor antibody (TRAb) tests, hypoechoic gland with increased vascularization at ultrasonography and diffuse uptake at thyroid scan (29). Eligibility criteria included serum Se concentration < 120 mcg/liter (13) and plasma VitD concentration < 30 ng/ml (30). Exclusion criteria were: age < 18 years or > 70 years, pregnancy and lactation, history or increased risk of fragility fractures (known osteoporosis, hyper/hypocalcemia, osteomalacia, sarcoidosis), type 1 diabetes, known immune or hematologic disorders, kidney failure, malabsorption/malnutrition, severe cardiac or lung diseases, active neoplasia, alcoholism, previous/ongoing treatment for hyperthyroidism, Se and/or VitD or multivitamin supplementation over the past three months, borderline/negative TRAb tests, baseline VitD ≥ 30 ng/ml and/or Se ≥ 120 mcg/l levels, intolerance to any of Se capsule excipients, contraindications to MMI treatment or inability to provide informed consent.

All participants signed a written informed consent form at the time of enrolment.

### Study Design

**Figure 1:**
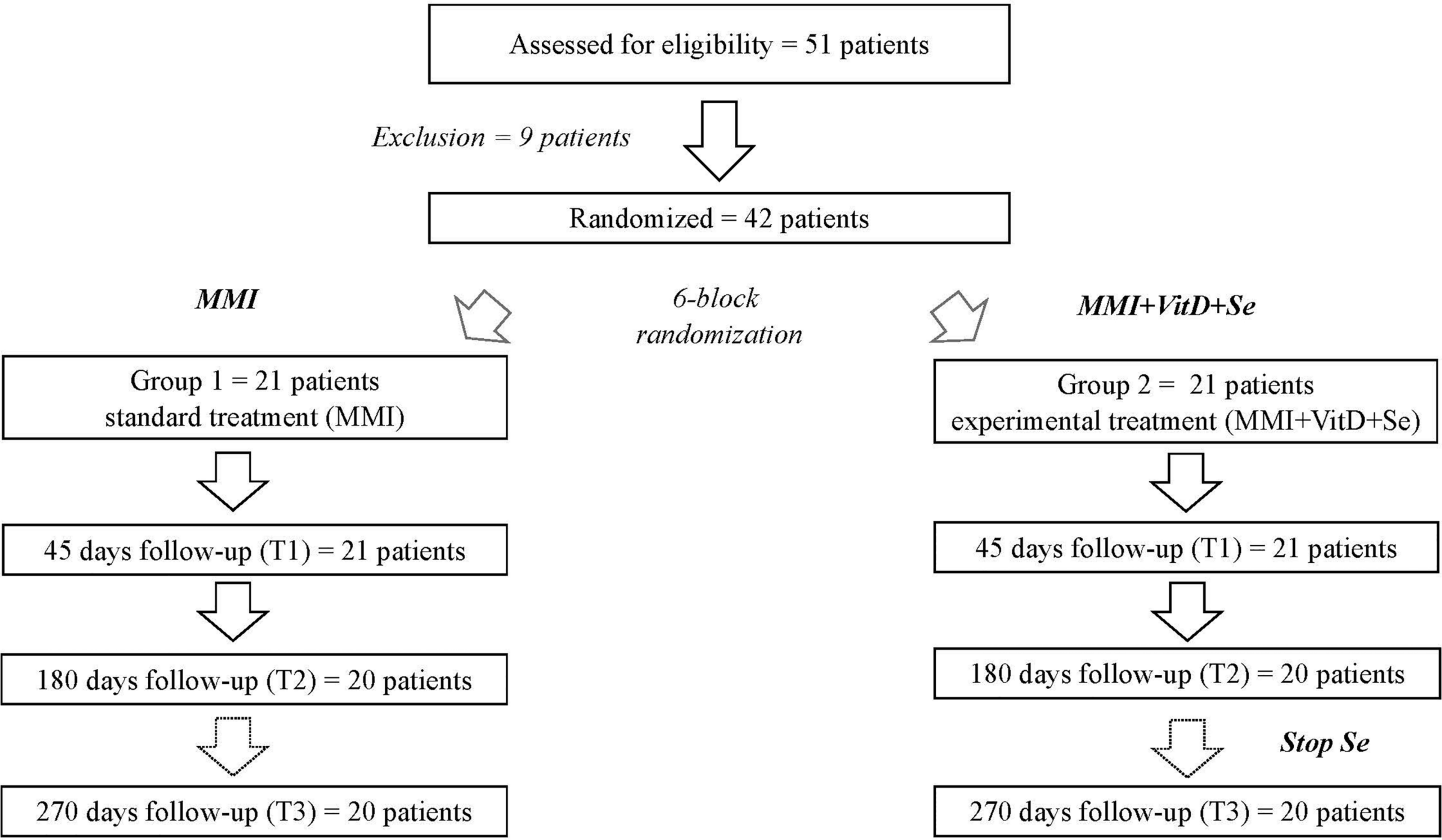
Flow-chart describing the design of the study.

This was a randomized, single-blinded, controlled, intervention trial. The RCT was approved by the local Ethics Committee (ASST dei Sette Laghi, Varese, registration number 92/2017) and by AIFA (Agenzia Italiana del Farmaco, measure SC 14282), and registered on EU Clinical Trials Register (EudraCT number 2017-005050-11). Starting with 001, a computer algorithm generated a six-block-size randomization list. Eligible patients were randomly assigned to treatment with MMI monotherapy (Group 1, MMI alone group) or MMI combined with Se and VitD (Group 2, intervention group), according to the randomization list (**Figure 1**). Each patient was assigned a unique sequential number (001, 002, and so on) that was recorded on a patient identification list alongside demographic data (name, date of birth). Biochemical and clinical data were collected at baseline, after 45 (T1), 180 (T2) and 270 (T3) days. At each study appointment, an unblinded investigator, who was not part of the team of endocrinologists responsible for the clinical work-up, filled out the Case Report Form (CRF module), which detailed the results of biochemical tests, handgrip strength performance and quality of life (QOL) questionnaires, along with ongoing doses of trial drugs (MMI, cholecalciferol and Se), and every dispensation and return of trial drugs. The results of the VitD and Se tests were not shared with RCT participants or the clinical team.

### Treatment

In both groups, starting daily MMI dose was established on baseline FT4 levels: 5-10 mg when serum FT4 levels were within 1.5-fold above the upper limit of normal (ULN); 15-20 mg for serum FT4 levels >1.5-2-fold above ULN; 30-40 mg for FT4 levels >2-3-fold above ULN (29). MMI dose was gradually tapered at routine outpatient visits, according to the titration method (29), as per our current clinical practice, by a team of endocrinologists who were not involved in the experiment as investigators and were unaware of each patient’s treatment group. Patients in the intervention group were given at each study visit a supply of Se and cholecalciferol that would last for the time between study appointments.

Supplementation consisted in 100 mcg/day of Se (1 tablet containing selenomethionine 83 mcg + selenium yeast 17 mcg, Syrel, Ibsa Farmaceutici Italia, Lodi) for 6 months, and cholecalciferol 7000 IU weekly (DIBASE, Abiogen Pharma, Pisa, oral solution vial 10.000 IU, 28 drops/week) for the trial duration, after a bolus dose calculated on baseline 25(OH)D levels (30) (DIBASE oral solution vial 300.000 IU, *una tantum* for 25(OH)D levels < 30 ng/ml, once a week for two consecutive weeks in case of 25(OH)D levels < 20 ng/ml, or once a week for three consecutive weeks in case of 25(OH)D levels < 10 ng/ml).

### Primary and Secondary Outcomes

The primary outcome was the between-arms difference in FT4 levels mean decrease from baseline to 180 days. Secondary outcomes included changes in biochemical markers (between-groups difference in the variation of FT4 levels after 45 and 270 days and the variations of FT3 and TRAb levels after 45, 180 and 270 days), clinical parameters (variation of handgrip strength performance) and QoL scores at 45, 180 and 270 days.

### Methods

Serum TSH, FT4 and FT3 concentrations were measured by electrochemiluminescence immunoassay (Analytical Unit for immunochemistry Cobas e801, Roche); reference ranges: TSH, 0.27-4.2 mU/liter (range of detection 0.005-100 mU/liter); FT4, 9.3-17 pg/ml (0.3-77 pg/ml); FT3, 2-4.4 pg/ml (0.1-13.6 pg/ml). TRAb levels were measured by second-generation radioreceptor assay (Thermofisher, Germany), normal value, <1 U/liter; upper limit of detection, 40 U/liter.

Serum Se levels were measured by a transversely-heated graphite atomizer furnace atomic absorption spectrometer (Analyst 600, PerkinElmer^®^, Waltham, USA), equipped with an electrodeless discharge lamp (PerkinElmer^®^); limit of detection (LOD), 10 mcg/liter, linear range (LR) 10-230 mcg/liter, inter-day coefficient of variation, 4.3% (12, 13). A normative sample of 150 healthy subjects yielded a mean Se value of 107.4 ± 12.2 mcg/liter (females 107.4 ± 12.2 mcg/liter vs. males 109.6 ± 15.5 mcg/liter, p=0.3; range 69.1- 104.3 mcg/l).

Plasma VitD concentration was measured by liquid chromatography/mass spectrometry (Shimadzu^®^ Nexera Liquid Chromatography modules coupled to Sciex^®^ Triple Quad 6500^+^ Mass Spectrometer) (30, 31); LOD 0.67 ng/ml, limit of quantification 2.25 ng/ml, LR 2.25–250 ng/ml, mean recovery rate 85-104%, intraassay coefficient of variation 3.2%, interassay coefficient of variation 3.4%. VitD status was categorized as “severe deficiency” in case of 25(OH)D levels ≤ 10.9 ng/ml, deficiency for values 11-19.9 ng/ml, insufficiency 20-29.9 ng/ml and sufficiency if values were ≥30 ng/ml (30). Normative values for our laboratory: 29 ± 12 ng/ml (range 6.1-30.8 ng/ml).

Thyroid volume was assessed by ultrasonography (ESAOTE MyLab, Esaote Spa) and determined using the ellipsoid formula: width (mm) x length (mm) x thickness (mm) x 0.52 x each lobe = volume (ml). In our area, the upper limit of the normal range is ≤18 ml in men and ≤14 ml in women (32).

The Clinical Severity Score (CSS) was applied to define GD severity at baseline, as previously described (32).

During a maximal isometric effort, handgrip strength (kg/s) was measured by Baseline^®^ hydraulic hand dynamometer (Fabrication Enterprises, Incorporated, White Plains, NY, USA). The mean value of three measurements over 2-minute intervals was considered for the analysis, as previously described (33).

Quality of life was assessed by self-administration of the validated Italian 39 items version of the ThyPRO (Thyroid-related Patient-Reported Outcome) questionnaire (34).

### Statistical Methods

**Figure 2:**
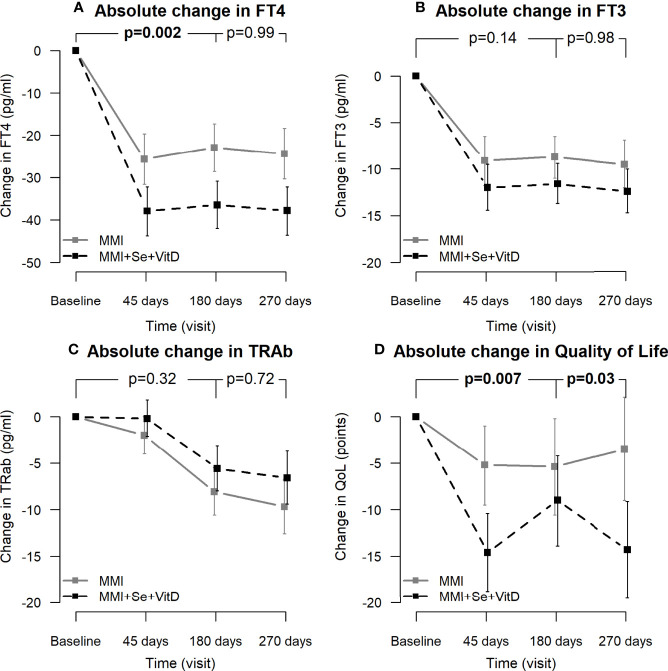
Absolute temporal changes of FT4 levels **(A)**, FT3 levels **(B)**, TRAb levels **(C)** and quality of life **(D)**. p=for the difference in time trends between the two groups. Baseline FT4, FT3, TRAb levels were included in the ANOVA model as covariates.

The sample size computation was based on n = 1000 simulation runs of a repeated-measure linear regression model, with FT4 as an outcome, treatment as exposure, and visit time and baseline FT4 as covariates. In simulations, distributional parameters for FT4 levels at baseline, 45, 180 and 270 days were taken from previous literature (27, 35, 36). We estimated that a study with 55 patients equally distributed in the two groups would have had an 80% probability to detect as statistically significant (alpha of 0.05) a between-arms mean difference in FT4 levels at 180 days of 3 pg/ml. We further assumed a drop-out rate of around 10%, yielding to a final sample size of 30 patients per treatment arm. We had to cease enrolment in February 2020, before recruiting was completed, due to the spread of the SARS-CoV-2 epidemic. The null hypothesis of no difference in time change between the study groups was tested using a Wald chi-square test with 4 degrees of freedom. Furthermore, we report the p-values for cross-sectional comparisons in FT4 mean values between groups at each visit (**Figure 2** and **Supplementary Figure 1A**). Similar longitudinal analyses were repeated for Se and 25(OH)D levels, as measures of treatment compliance (**Table 2**) and for secondary outcomes, such as serum FT3 (**Figure 2B**) and, TRAb levels (**Figure 2C**), QoL composite score and subscales (**Figure 2D** and Supplementary Table) and handgrip strength. Baseline levels of FT4, FT3, and TRAb were included as covariates in longitudinal and cross-sectional analysis. The statistical analyses were performed using the SAS software 9.4 release.

## Results

Fifty-one consecutive newly diagnosed GD patients were screened for enrolment during the study period. Nine patients were excluded, six due to normal VitD levels, one for multivitamin supplementation, one for age < 18 years and one for borderline TRAb values (**Figure 1**). Baseline demographic and clinical features of the 42 enrolled patients (37 women and 5 men, aged 45.8 ± 10.3 years) are shown in **Table 1**. According to the CSS, GD was severe in 44% of cases, moderate in 36.5% and mild in 19.5%, and patients in the intervention group had the more severe disease at baseline. Serum Se levels (mean value: 93 ± 10.2 mcg/liter) were ≤ 90 mcg/liter in 24 patients and < 110 mcg/liter in the remaining 18 patients. Baseline 25(OH)D concentrations (mean value: 19.2 ± 7.3 ng/ml) were consistent with severe deficiency in 6 patients, deficiency in 20 and insufficiency in 16 patients.

**Table 1:** Main demographic and clinical features of the 42 Graves’ disease patients enrolled, in the whole group and divided by arms of treatment.

Parameters	Whole sample	MMI	MMI+Se+VitD	p
***Number***	**42**	**21**	**21**	**-**
***Age, years***	45.8 ± 10.3	47.7 ± 11.4	45.8 ± 9.3	0.55
***Sex, women n (%)***	37 (87.8)	19 (95)	18 (81)	0.17
***Smokers, n (%)***	16 (39)	5 (25)	11 (52.4)	0.07
***CSS***				
*Mild, n (%)*	8 (19.5)	4 (20)	4 (19.1)	
*Moderate, n (%)*	15 (36.5)	13 (60)	3 (14.3)	**0.004**
*Severe, n (%)*	19 (44)	4 (20)	14 (66.7)	
***ALT, U/l***	25.9 ± 8.3	26.3 ± 9.9	25.6 ± 6.9	0.79
***AST, U/l***	35 ± 18.6	36.4 ± 24.2	33.7 ± 11.5	0.66
***Calcium, mg/dl***	9.6 ± 0.3	9.7 ± 0.3	9.7 ± 0.4	0.60
***BMI, kg/m^2^***	22.9 ± 3.2	23.8 ± 3.5	22 ± 2.8	0.08
***DBP, mmHg***	78 ± 8.3	76.8 ± 8.6	79.3 ± 8	0.33
***SBP, mmHg***	128.04 ± 16.1	126 ± 16.3	130 ± 16.1	0.43
***HR, bpm***	91.4 ± 15.6	89.1 ± 16.8	93.7 ± 14.6	0.35

Data were reported as mean ± standard deviation or frequency (percentage). p=p-value testing baseline differences between the two groups. MMI=methimazole alone group; MMI+Se+VitD=intervention group treated with methimazole, selenium and cholecalciferol. ALT, alanine aminotransferase; AST, aspartate aminotransferase; BMI, body mass index; DBP, diastolic blood pressure; CSS, Clinical Severity Score; HR, heart rate; SBP, systolic blood pressure. BMI was calculated using the formula weight/square height^2^. Bold values for p values means statistically significant difference.

Randomization was balanced for demographic and anthropometric features. Patients in the intervention group had a more severe form of disease (**Table 1**) and worst QoL scores at baseline. These differences were appropriately considered during data elaboration by including the baseline values as covariates. Each group had one patient who was lost to follow-up. As a result, 40 subjects had complete data at 180 days. Starting daily MMI dose ranged between 15 and 30 mg (MMI group 19.3 ± 8.9 mg/day vs. intervention group 23.6 ± 9.8 mg/day; p = 0.15). After 30 days thyroid function was checked to adjust MMI dose, as per our current clinical practice. At 45, 180 and 270 days, the mean MMI dose did not differ between the two groups, nor did self-reported treatment compliance. No relevant adverse events occurred. At 45 and 180 days, serum Se concentrations increased significantly in the intervention group but not in the MMI group, with only the supplemented group achieving optimum concentrations (**Table 2**). No case of selenosis occurred. Three months after Se discontinuation, serum Se levels remained significantly higher in the intervention group compared to baseline and to MMI alone group (**Table 2**).

**Table 2:** Temporal variation of mean vitamin D (VitD) and selenium (Se) levels in the two groups of treatment at different temporal points.

MMI (n = 20)			MMI+Se+VitD (n = 20)			
	*mean*±SE	*Δ (*CI *95%)*	*mean*±SE	*Δ (* CI *95%)*	p*	p**
***Se, mcg/l***						
*baseline*	93.2±3.9	–	92.3±3.8	–		0.87
*45 days*	96.2±3.9	3 (-6.9;12.9)	142.1±3.8	49.9 (40.2; 59.5)	**0.001**	**0.0001**
*180 days*	96.5±4.7	3.4 (-7.5; 14.2)	164.7±3.9	72.4 (61.9; 83)	**0.001**	**0.0001**
*270 days*	98.8±4.9	5.6 (-6.7;17.9)	123.7±4.7	31.4 (19.5; 43.3)	–	**0.0004**
***VitD, ng/ml***						
*baseline*	21.2±2.2	–	16.5±2.1	–		0.133
*45 days*	19.6±2.2	-1.6 (-7; 4.2)	53.9±2.1	37.8 (32.1; 43.4)	**0.001**	**0.0001**
*180 days*	20.5±2.3	-0.6 (-6.7; 5.4)	32.8±2.2	16.7 (10.8; 22.6)	**0.001**	**0.0002**
*270 days*	20.9±2.6	-0.3 (-6.9; 6.2)	30.7±2.3	16.7 (10.8; 22.6)	**0.001**	**0.008**

Temporal variation of Selenium (Se) and Vitamin D (VitD) levels in the 40 patients which have complete data during follow-up. Data were reported as mean ± standard error (SE). SE = Standard Error; CI 95%, confidence interval; MMI, methimazole; Se, Selenium; VitD, Vitamin D; Δ (IC 95%): variations from baseline to 45, 180 and 270 days. p*= p-value for interaction between time course and treatment group; p**= p-value for intergroup comparison. Bold values for p values means statistically significant difference.

Plasma VitD levels increased only in the intervention group (**Table 2**), whereas they remained stable or slightly decreased in the MMI group at 180 days. There was a significant difference in VitD levels and in the degree of VitD change between the two groups at 45 and 180 days. VitD levels in the intervention group reached their maximum after 45 days in response to the cholecalciferol loading dose; after 6 months of treatment, all patients in the group had sufficient VitD levels, which remained within the normal range after 9 months. Therapy was well tolerated, with no cases of hypercalcemia. **Figure 2** and **Supplementary Figure 1** show that MMI treatment significantly lowered serum FT4 levels after 45 and 180 days in the MMI group (respectively: -25.7 pg/ml, -31.6 to -19.7 pg/ml and -22.9 pg/ml, -28.6 to -17.3 pg/ml, but significantly more in the intervention group (respectively -37.9 pg/ml, -43.7 to -32.2 pg/ml and -36.5 pg/ml, -42 to -30.9 pg/ml, with significant greater variation from baseline (including baseline FT4 levels as covariate the between-arms mean difference for FT4 variation was 12.2 pg/ml, p=0.002). When compared to the MMI alone group, the intervention group’s FT4 levels had changed more from baseline also at 270 days (-37.8 pg/ml, -43.6 to -32.1 pg/ml vs. -24.4 pg/ml, -30.3 to -18.4 pg/ml vs. -24.4 pg/ml, -30.3 to -18.4 pg/ml *vs.* -24.4 pg/ml), but the two groups had a similar trend of variation from 180 to 270 days (p 0.99). Despite the intervention group having more severe hyperthyroidism at baseline (p-value t-test for independent variables 0.001), mean FT4 values at 45 days (mean FT4 levels 9.1 pg/ml in the intervention group vs. 11.3 pg/ml in MMI alone group, p-value t-test = 0.44 for independent variables), 180 days (10.6 pg/ml vs. 14 pg/ml, p = 0.23) and 270 days (mean FT4 levels 9,2 pg/ml in the intervention group vs. 12.6 pg/ml in MMI alone group, p-value t-test = 0.28 for independent variables) were similar comparing the two groups, and within the range of normal values (**Supplementary Figure 1**). Serum FT3 and TRAb levels had a similar decrease comparing the two groups (**Figure 2** and **Supplementary Figures 1B, C**). Intervention group had a significantly greater improvement in QoL composite scores compared to MMI group (p=0.007 for baseline-180 days and p=0.03 for 180-270 days interval), both in the short term (at 45 days -14.6, CI 95% -18.8 to -10.4 vs. -5.2, CI 95% -9.5 to -1, p = 0.007) and long-term (-14.3, CI 95% -19.5 to -9.1 *vs.* -3.5, CI95% -9 to 2.1, p 0.003) (**Figure 2** and **Supplementary Figure 1D**). Cognition and social life impairment showed the greatest advantages (at 45 days: intervention group *vs.* MMI group: cognition scale -1.9, CI 95% -2.9 to -0.9 vs. -0.2, CI 95% -1.3 to 0.8, p = 0.04; social life impairment scale -1.2, CI 95% -2 to -0.4 vs.. 0, CI 95% -1 to 0.9, p 0.05); the additional benefit of the intervention continued after Se suspension (cognition scale at 270 days: mean score 1.3 in the intervention group vs. 3 in MMI alone group, p 0.01; entity of variation, respectively 0.6, CI 95%, -0.7 to 1.8 vs. -1.6, CI95% -2.7 to -0.4) (**Supplementary Table 1**). Systolic blood pressure and heart rate levels significantly improved in both groups during treatment compared to baseline with a similar trend in the two groups (data not shown). Handgrip strength improved over time in the intervention and MMI-alone groups, compared to baseline, with no significant differences between the two arms of treatment (**Supplementary Table 2**).

## Discussion

Data on VitD supplementation in GD patients treated with ATDs are scant, although two studies suggested that combination therapy was associated with a slightly greater reduction in serum free thyroid hormones compared to MMI monotherapy (27, 28). No information on Se status in these studies was provided. Other studies evaluated the effect of Se addition to MMI in the treatment of Graves’ hyperthyroidism, but results were conflicting, as either no beneficial effect in terms of control/relapse of hyperthyroidism (25) or a prompter control of thyroid hyperfunction were reported (19). A recent meta-analysis of ten trials (796 patients) showed a beneficial effect of Se supplementation on biochemical parameters of thyroid function at 6 months, but not at 9 months (22).

It is worth noting that the optimal range of Se is likely to be narrow, and therefore Se supplementation should be approached with caution. According to the PRECISE trial, 300 mcg/day of Se supplementation given for five years in a country with low Se status increased all-cause mortality 10 years later (23). In another study, supplementing with Se at lower doses (160 mcg/day) for 6 months led to Se levels beyond the approved reference range (24). None of the above studies provided information on VitD status. In the present RCT, we investigated whether, in newly diagnosed GD patients with concomitant low Se and VitD levels, a 6-month supplementation with both micronutrients might facilitate the restoration of euthyroidism during ATD treatment. In our study, baseline Se levels were low (but not very low), while VitD levels were clearly subnormal. Se supplementation was stopped after 6 months to avoid Se excess, and Se levels remained within the therapeutic range for the whole study duration (including evaluation at 270 days). The major finding of our study was that supplementation favours a significantly better control of hyperthyroidism, both at short-term (45 days) and long-term (180 and 270 days) assessments. In addition, a faster decrease in FT4 levels in the supplemented group was observed, permitting a greater reduction of the MMI dose after 30 days of treatment compared to the MMI monotherapy group, so that the mean daily dose of MMI at 45 days was similar in the two groups (data not shown). This effect was clearly evident in serum FT4, which is currently recognized as the only reliable marker for monitoring response to ATD (2). Although there was also a trend in serum FT3, it did not achieve statistical significance. This might be explained by the interference of multiple factors (including short FT3 life, lower precision of laboratory tests) and by the upregulations of type 2 deiodinase in hyperthyroidism (35, 37). The two groups of study reported a similar decrease in TRAb levels. Because the intervention group’s FT4 drop was greater, a similar trend for TRAb levels was predicted. However, this group had a more severe condition, thus, achieving mean TRAb levels similar to those of the MMI group, might supports the favorable impact of the supplementation. It should be recalled that TRAb synthesis is the culmination of a multi-step pathogenetic process that includes innate and cellular immunity in addition to the humoral counterpart (38, 39). The combined treatment may have a stronger effect on cellular and innate immunity than on humoral immunity. This hypothesis is supported by the effects of VitD and Se on the proliferation, differentiation, and recruitment of dendritic cells, T helper cells, and T regulatory cells (7, 10).

A significantly greater and positive impact on the QoL was also observed, as, according to the results of the ThyPRO questionnaire, a more pronounced improvement in both the composite score and in social and cognition subscales was achieved in the supplemented group. Besides the benefits of a better control of hyperthyroidism, explanations should consider a potential direct effect of VitD (40, 41) and Se (42) on mood. Interestingly, the cognition subscale reached a better score in the long term (270 days) in the intervention group than the MMI alone group, suggesting a leading role of cholecalciferol supplementation.

Discrepant results in the literature on the effect of Se supplementation in patients with Graves’ hyperthyroidism might be related to the type and duration of Se supplementation, as well as to baseline Se levels. At variance with previous studies (19), in our trial Se levels were screened before enrollment to ascertain the necessity of supplementation; because Se concentrations ≥ 120 mcg/liter correspond to the point at which selenoprotein P reached a plateau in response to increased Se dosages (13), we used this cutoff to determine Se sufficiency.

An alternative explanation for the conflicting results provided by Se supplementation might be that studies reporting negative results included patients with VitD insufficiency/deficiency, and the latter is a more important micronutrient to be supplemented. In other words, it might be postulated that optimization of VitD status was the main responsible for the positive result of our study on hyperthyroidism control. In addition, it is conceivable that VitD and Se may synergize, regulating two major biological events: immune homeostasis and oxidative metabolism. A factorial study randomizing patients in four arms (Se+placebo+MMI vs. placebo+VitD+MMI vs. Se+VitD+MMI vs. MMI alone) was not feasible in our single Centre due to the large number of cases required to obtain an adequate sample size. Recently, supplementation with VitD in Hashimoto’s thyroiditis proved to be more effective in reducing antithyroid antibody levels in patients previously treated with Se than in non-pretreated patients, suggesting that Se intake might enhance the effects of VitD supplementation on immune regulation (36).

The major strength of our study is the novelty of the association of Se and VitD to MMI in the management of Graves’ hyperthyroidism. Other strengths included strict enrolment criteria, screening of VitD and Se status before enrollment, centralization of all tests which were performed by highly specialized teams using gold standard techniques, and direct assessment of patients. One of the study’s major limitations was that the two randomized groups were not balanced in terms of GD severity at baseline, as was the fact that the sample size was smaller than the planned target. Both these limitations might be explained by the premature interruption of recruitment, due to the SARS-CoV-2 pandemic spread. However, in an attempt to overcome this potential bias, baseline FT4, FT3 and TRAb levels were included as a covariate in the analysis.

In conclusion, the results of this study in a cohort of newly diagnosed, consecutive GD patients, with borderline low Se levels and VitD insufficiency, suggest that i) Se and VitD supplementation facilitates restoration of euthyroidism during MMI treatment; ii) Se and VitD supplementation boosts the improvement of QoL during MMI treatment. It is reasonable to propose that Se and VitD status be assessed at diagnosis of GD, and Se and VitD supplementation be offered at adequate and safe dosages if even a slight deficiency of these micronutrients is found.

## Data Availability Statement

The raw data supporting the conclusion of the article will be made availble upon reasonable request.

## Ethics Statement

The studies involving human participants were reviewed and approved by Comitato Etico ASST Sette Laghi, Varese. mail: comitato.etico@asst-settelaghi.it. The patients/participants provided their written informed consent to participate in this study.

## Author Contributions

All the authors were involved in manuscript drafting. DG, EP, LB, GV, LM, and AP (11th Author) designed the study protocol. GV and PL performed statistical analysis. AN, ML, AP (11th Author), AP (12th Author), and PM were responsible for chemistry (thyroid hormones, thyroid antibodies, 25(OH) D levels tests). CP elaborated and performed Se analysis. All authors contributed to the article and approved the submitted version.

## Funding

DG was supported by a University of Insubria PhD scholarship in Experimental and Translational Medicine. EP was supported by Fondo di Ateneo per la Ricerca FAR 2018, University of Insubria. AB is recipient of a research grant funded by the Italian Association for Cancer Research (AIRC-MFAG, ID 22818) and a research grant funded by the Cariplo Foundation (ID 2019-1609) and is funded by the Ricerca Corrente, IRCCS MultiMedica. MG is a participant to the PhD course in Life Sciences and Biotechnology at the University of Insubria and is funded by a fellowship within the PRIN 2017 grant 2017NTK4HY.

## Conflict of Interest

The authors declare that the research was conducted in the absence of any commercial or financial relationships that could be construed as a potential conflict of interest.

## Publisher’s Note

All claims expressed in this article are solely those of the authors and do not necessarily represent those of their affiliated organizations, or those of the publisher, the editors and the reviewers. Any product that may be evaluated in this article, or claim that may be made by its manufacturer, is not guaranteed or endorsed by the publisher.

## Supplementary Material

Supplementary Table 1 Temporal changes of QoL scales investigated by 39 items Thyroid-specific quality of life patient-reported outcome questionnaire (ThyPRO). p-value*= p-value for interaction between time course and treatment group (period of evaluation: 0-6 months and 6-9 months); p-value**= p-value for intergroup comparison. social, social life impairment; goiter, symptoms related to the presence of enlarged thyroid gland; hyperthyroid, symptoms related to hyperthyroidism, ThyPRO, Thyroid-specific quality of life patient-reported outcome questionnaire.

Supplementary Table 2 Temporal changes of handgrip strength test (kg/s). p-value*= p-value for interaction between time course and treatment group (period of evaluation: 0-6 months and 6-9 months); p-value**= p-value for intergroup comparison.

Supplementary Figure 1 Mean variation of FT4 levels **(a)**, FT3 levels **(b)**, TRAb levels **(c)** and quality of life **(d)**.
