# Supplementary material for: Add-On Effect of Selenium and Vitamin D Combined Supplementation in Early Control of Graves’ Disease Hyperthyroidism During Methimazole Treatment
Source: Front Endocrinol (Lausanne). 2022 Jun 15;13:886451. doi: 10.3389/fendo.2022.886451 (PMC9240752; doi:10.3389/fendo.2022.886451)
Supplement: Supplementary Table 1 — Temporal changes of QoL scales investigated by 39 items Thyroid-specific quality of life patient-reported outcome questionnaire (ThyPRO). p-value*= p-value for interaction between time course and treatment group (period of evaluation: 0-6 months and 6-9 months); p-value**= p-value for intergroup comparison. social, social life impairment; goiter, symptoms related to the presence of enlarged thyroid gland; hyperthyroid, symptoms related to hyperthyroidism, ThyPRO, Thyroid-specific quality of life patient-reported outcome questionnaire. [file Table_1.docx]

|  | **Group 1 (MMI)** | | **Group 2 (MMI+Se+VitD)** | |  |  |  |
| --- | --- | --- | --- | --- | --- | --- | --- |
|  | *mean* | *Δ (CI 95%)* | *mean* | *Δ (CI 95%)* | *p-value*****^0-6/6-9^*** | *p-value*** |  |
| **Goiter** |  |  |  |  |  |  |  |
| *baseline* | 1.7 | - | 2.3 | - |  | 0.33 |  |
| *45 days* | 1.4 | -0.3 (-1.3; 0.6) | 1.1 | -1.2 (-2.1; -0.3) | 0.18/0.25 | 0.56 |  |
| *180 days* | 1.8 | 0.2 (-1; 1.3) | 1 | -1.3 (-2.3; -0.2) |  | 0.15 |  |
| *270 days* | 2.6 | 0.9 (-0.3;2.1) | 0.9 | -1.4 (-2.5;-0.2) |  | **0.01** |  |
| **Hyperthyroid** |  |  |  |  |  |  |  |
| *baseline* | 7.1 | - | 7.3 | - |  | 0.76 |  |
| *45 days* | 3.9 | -3.2 (-4.5; -2) | 2.9 | -4.4 (-5.6; -3.2) | 0.29/0.58 | 0.18 |  |
| *180 days* | 3.3 | -3.8 (-5.2; -2.4) | 2.2 | -5.1 (-6.4; -3.8) |  | 0.13 |  |
| *270 days* | 3.3 | -3.8 (-5.3;-2.3) | 1.7 | -5.6( -7.1;-4.2) |  | **0.04** |  |
| **Tiredness** |  |  |  |  |  |  |  |
| *baseline* | 4.7 | - | 4.9 | - |  | 0.62 |  |
| *45 days* | 3.9 | -0.8 (-1.7; 0) | 3.6 | -1.4 (-2.2; -0.5) | 0.54/0.36 | 0.53 |  |
| *180 days* | 3.8 | -0.9 (-1.8; 0) | 4.1 | -0.9 (-1.7; 0) |  | 0.52 |  |
| *270 days* | 3.7 | -1 (-2;-0.1) | 3.4 | -1.5 (-2.4;-0.6) |  | 0.59 |  |
| **Cognition** |  |  |  |  |  |  |  |
| *baseline* | 2.5 | - | 2.8 | - |  | 0.58 |  |
| *45 days* | 2.3 | -0.2 (-1.3; 0.8) | 0.9 | -1.9 (-2.9; -0.9) | **0.02/0.02** | **0.03** |  |
| *180 days* | 2.4 | 0 (-1.3; 1.2) | 2.5 | -0.4 (-1.5; 0.8) |  | 0.96 |  |
| *270 days* | 3 | 0.6 (-0.7;1.8) | 1.3 | -1.6 (-2.7;-0.4) |  | **0.01** |  |
| **Anxiety** |  |  |  |  |  |  |  |
| *baseline* | 3.5 | - | 3.7 | - |  | 0.74 |  |
| *45 days* | 2 | -1.5 (-2.6; -0.3) | 1 | -2.8 (-3.9; -1.6) | 0.21/0.29 | 0.05 |  |
| *180 days* | 2.3 | -1.1 (-2.4; 0.1) | 1.8 | -1.9 (-3; -0.8) |  | 0.34 |  |
| *270 days* | 2.8 | -0.7 (-1.9;0.5) | 1.4 | -2.3 (-3.4;-1.2) |  | **0.03** |  |
| **Depression** |  |  |  |  |  |  |  |
| *baseline* | 3.7 | - | 3.8 | - |  | 0.82 |  |
| *45 days* | 3.6 | -0.2 (0.9; 0.6) | 2.8 | -1 (-1.8;-0.2) | 0.18/0.46 | 0.11 |  |
| *180 days* | 3 | -0.7 (-1.6; 0.2) | 3.1 | -0.2 (-1.6; 0.2) |  | 0.79 |  |
| *270 days* | 3.1 | -0.7 (-1.6;0.3) | 2.8 | -1.1 (-2;-0.2) |  | 0.54 |  |
| **Susceptibility** |  |  |  |  |  |  |  |
| *baseline* | 5.2 | - | 5.7 | - |  | 0.41 |  |
| *45 days* | 4.6 | -0.6 (-1.5; 0.4) | 3.7 | -2 (-2.9; -1.1) | 0.11/0.40 | 0.12 |  |
| *180 days* | 4.9 | -0.2 (-1.4; 0.9) | 4.5 | -1.2 (-2.2; -0.1) |  | 0.49 |  |
| *270 days* | 4.9 | -0.3 (-1.5;0.9) | 3.9 | -1.8 (-2.9;-0.7) |  | 0.11 |  |
| **Impaired social life** |  |  |  |  |  |  |  |
| *baseline* | 1.3 | - | 2 | - |  | 0.11 |  |
| *45 days* | 1.3 | -0.1 (-0.9; 0.7) | 0.6 | -1.4 (-2.2; -0.6) | **0.05/**0.27 | 0.19 |  |
| *180 days* | 1.3 | 0 (-1; 0.9) | 0.8 | -1.2 (-2; -0.4) |  | 0.38 |  |
| *270 days* | 1.3 | 0 (-1;1) | 0.3 | -1.8 (-2.7;-0.9) |  | **0.03** |  |
| **Impaired daily life** |  |  |  |  |  |  |  |
| *baseline* | 2.6 | - | 3 | - |  | 0.53 |  |
| *45 days* | 1.8 | -0.8 (-1.9; 0.4) | 1 | -2 (-3; -0.9) | 0.13/0.12 | 0.26 |  |
| *180 days* | 1.6 | -0.9 (-2.3; 0.5) | 2 | -1 (-2.2; 0.3) |  | 0.60 |  |
| *270 days* | 1.6 | -1 (-2.4;0.4) | 0.7 | -2.3 (-3.6;-1) |  | 0.26 |  |
| **Cosmetic compliants** |  |  |  |  |  |  |  |
| *baseline* | 2.3 | - | 2.6 | - |  | 0.68 |  |
| *45 days* | 2.3 | -0.1 (-1.4; 1.2) | 2.2 | -0.4 (-1.6; 0.9) | 0.73/0.78 | 0.89 |  |
| *180 days* | 2 | -0.3 (-1.8; 1.1) | 1.5 | -1.1 (-2.4; 0.2) |  | 0.49 |  |
| *270 days* | 2.2 | -0.1 (-1.5;1-4) | 1.5 | -1.1 (-2.5;0.2) |  | 0.30 |  |
|  |  |  |  |  |  |  |  |
|  |  |  |  |  |  |  |  |

p-value*= p-value for interaction between time course and treatment group (period of evaluation: 0-6 months and 6-9 months); p-value**= p-value for intergroup comparison.

Abbreviations: social, social life impairment; goiter, symptoms related to the presence of enlarged thyroid gland; hyperthyroid, symptoms related to hyperthyroidism, ThyPRO, Thyroid-specific quality of life patient-reported outcome questionnaire.
