# Supplementary material for: Add-On Effect of Selenium and Vitamin D Combined Supplementation in Early Control of Graves’ Disease Hyperthyroidism During Methimazole Treatment
Source: Front Endocrinol (Lausanne). 2022 Jun 15;13:886451. doi: 10.3389/fendo.2022.886451 (PMC9240752; doi:10.3389/fendo.2022.886451)
Supplement: Supplementary Table 2 — Temporal changes of handgrip strength test (kg/s). p-value*= p-value for interaction between time course and treatment group (period of evaluation: 0-6 months and 6-9 months); p-value**= p-value for intergroup comparison. [file Table_2.docx]

|  | **Group 1 (MMI)** | | **Group 2 (MMI+Se+VitD)** | |  |  |  |
| --- | --- | --- | --- | --- | --- | --- | --- |
|  | *mean* | *Δ (CI 95%)* | *mean* | *Δ (CI 95%)* | *p-value*****^0-6/6-9^*** | *p-value*** |  |
| **Hand Grip (kg/s)** |  |  |  |  |  |  |  |
| *baseline* | 23 | - | 20 | - |  | 0.87 |  |
| *45 days* | 22 | 2.1 (-0.1; 4.2) | 22.3 | 2.4 (0.5; 4.4) | 0.17/0.19 | 0.92 |  |
| *180 days* | 25.6 | 5.5 (2.9; 8) | 23 | 3.1 (0.8; 5.4) |  | 0.06 |  |
| *270 days* | 21.5 | -1.4 (-4.6;-1.7) | 22.5 | 1.6 (-0.3;3.4) |  | 0.3 |  |

p-value*= p-value for interaction between time course and treatment group (period of evaluation: 0-6 months and 6-9 months); p-value**= p-value for intergroup comparison.
