# Supplementary figures and images for: Add-On Effect of Selenium and Vitamin D Combined Supplementation in Early Control of Graves’ Disease Hyperthyroidism During Methimazole Treatment
Source: Front Endocrinol (Lausanne). 2022 Jun 15;13:886451. doi: 10.3389/fendo.2022.886451 (PMC9240752; doi:10.3389/fendo.2022.886451)

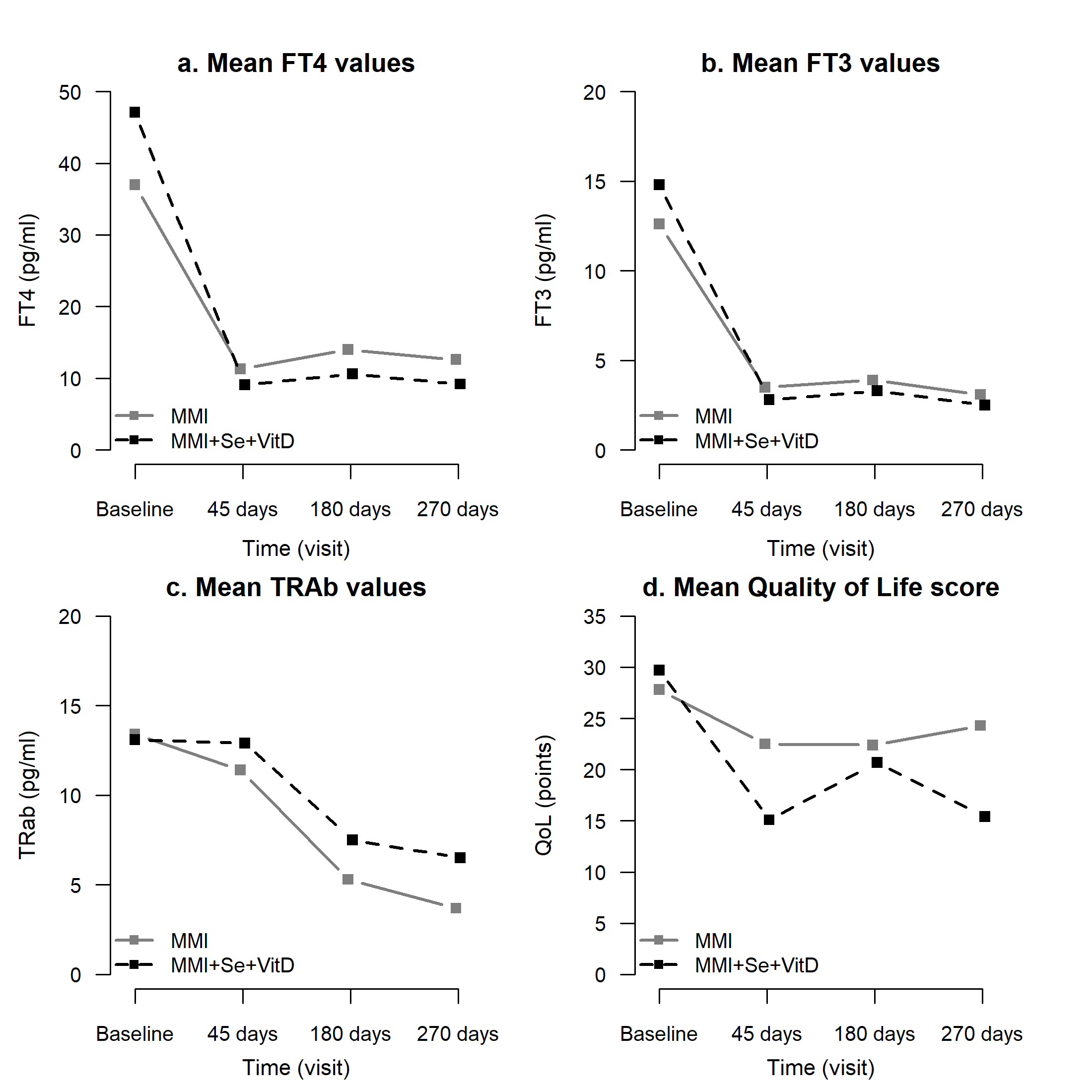

Supplement: Supplementary Figure 1 — Mean variation of FT4 levels (a), FT3 levels (b), TRAb levels (c) and quality of life (d). [file Image_1.tif]
